# Anterior Corpectomy and Vertebroplasty With Carbon-Polyetheretherketone (PEEK) Cage for Invasive Spinal Meningioma

**DOI:** 10.7759/cureus.90725

**Published:** 2025-08-22

**Authors:** Brian Hall, Parker Anderson, Lana D Christiano

**Affiliations:** 1 Neurosurgery, West Virginia School of Osteopathic Medicine, Charleston, USA; 2 Neurological Surgery, West Virginia School of Osteopathic Medicine, Lewisburg, USA; 3 Neurosurgery, Charleston Area Medical Center, Charleston, USA

**Keywords:** case report, corpectomy, peek, spinal implant, spinal meningioma, vertebroplasty

## Abstract

Intradural extramedullary tumors constitute a significant burden of primary spinal neoplasms. These include spinal meningiomas, the minority of which are located ventral to the cord. This report presents anterior corpectomy with Simpson Grade II resection of a ventrally located spinal meningioma at C3, followed by vertebral body replacement with a polyetheretherketone (PEEK) cage. The composition of this implant is of particular importance to this case, as it is necessary to monitor for the common event of meningioma recurrence. A 73-year-old female presented with left-sided lower extremity numbness and upper motor neuron findings. Imaging of the cervical spine revealed a large intradural extramedullary mass consistent with a meningioma, causing displacement of the cord to the left. An anterior cervical discectomy and fusion approach was used for C3 corpectomy and tumor resection, and a carbon-PEEK implant was used for spinal stabilization. The procedure continued in an uncomplicated fashion, and the transient dysphagia experienced by the patient resolved swiftly with supportive care. At a two-year follow-up, the patient remains asymptomatic and shows no evidence of meningioma recurrence. PEEK's resilience and radiolucency proved instrumental in ensuring the stability of the construct, as well as facilitating follow-up imaging of the cervical spine without artifacts. This case demonstrates the use of the anterior approach for corpectomy with a carbon-PEEK implant for spinal stabilization in the context of a ventrally located spinal meningioma, a strategy that ensured maximal safe resection of the lesion and permitted detailed postoperative surveillance for tumor recurrence. The success of this surgery and the patient's postoperative course highlight the critical role of a tailored surgical strategy and advanced biomaterials in optimizing outcomes for complex spinal neoplasms.

## Introduction

Intradural extramedullary tumors account for nearly two-thirds of primary spinal neoplasms and include meningiomas, schwannomas, and neurofibromas, each presenting unique diagnostic and therapeutic challenges. Spinal meningiomas constitute 25-30% of intradural extramedullary lesions but only 1.2-12% of all meningiomas, with 15-20% positioned ventral to the cord and the remainder dorsal or dorsolateral [1,2]. These tumors are known to cause spinal cord compression and require excision for definitive treatment. This is achieved through various approaches, depending on the location. An anterior approach excels for ventral cervical tumors, where the fascial planes offer free mobilization of structures anterior to the spine; however, it proves impractical in the thoracic spine, where thoracic structures may obstruct access. Surgical planning hinges on approach selection, stabilization, and follow-up imaging, striking a balance between tumor removal and preservation of neurological function and structural integrity.

This case involves the resection of a ventral C3 meningioma via an anterior approach, accompanied by corpectomy, followed by vertebral replacement using a polyetheretherketone (PEEK) implant, selected for its biocompatibility, resilience, and radiolucency, which is crucial for monitoring recurrence [3,4]. The anterior corridor enabled direct decompression, while the PEEK implant mitigated radiologic artifacting. The case highlights the tailored integration of technique and material choice in effectively managing such neoplasms.

## Case presentation

**Figure 1:**
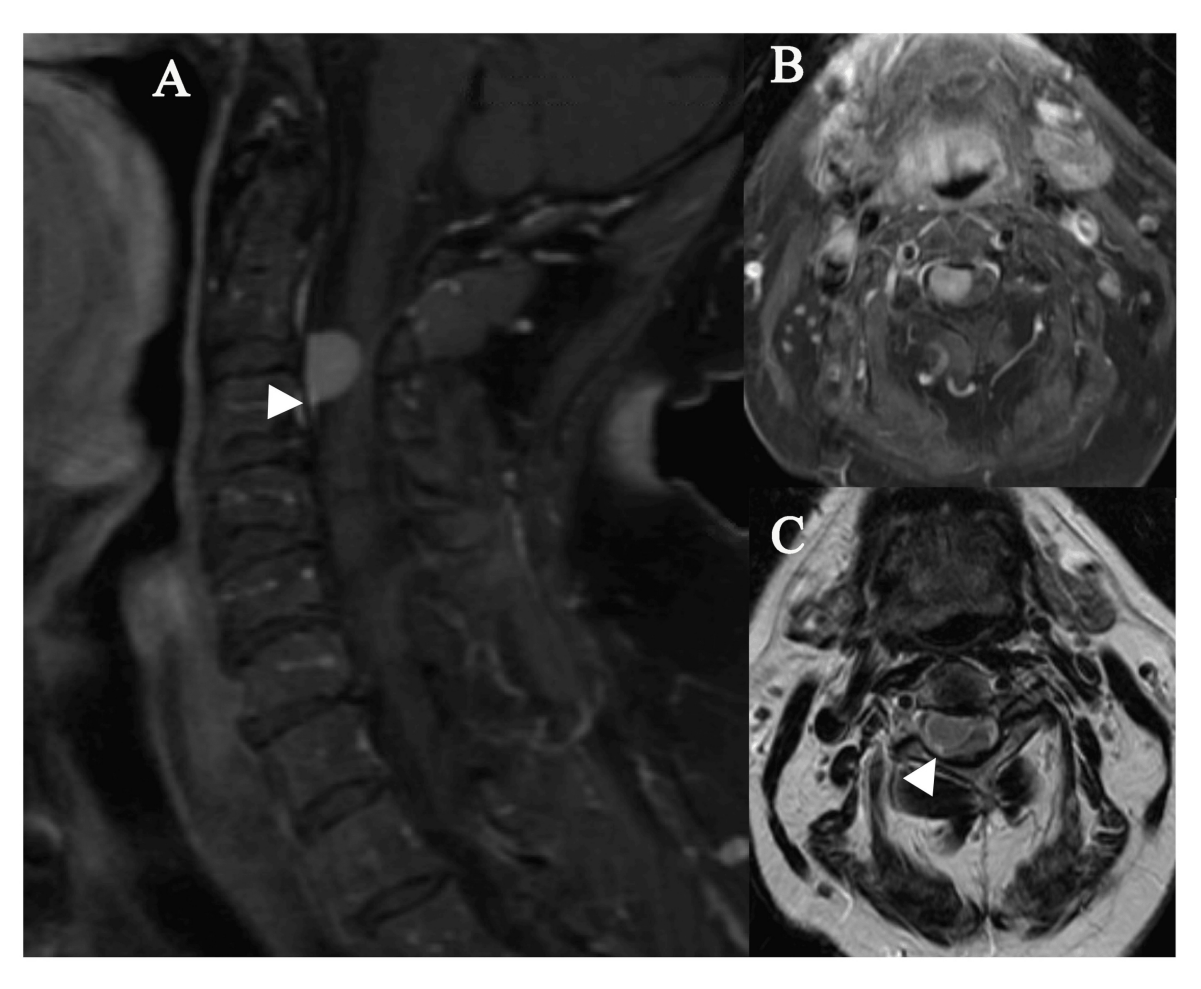
Preoperative MRI in multiple views (A) Sagittal post-gadolinium MRI demonstrating the ventral location of the tumor with significant canal compromise. The triangle denotes the dural tail, a pathognomonic feature of meningioma. (B) Axial post-gadolinium MRI demonstrating the ventral location and canal compromise. (C) Axial T2 sequence: the triangle denotes the border between the meningioma to the top left of the triangle and the spinal cord, which is to the bottom and right of the triangle. The spinal cord is significantly compressed and displaced dorsally and toward the patient's left.

A 73-year-old female presented with left lower extremity numbness following a recent fall with a fractured metatarsal. Examination revealed myelopathic signs, including a positive Hoffman reflex on the left and a brisk 3+ deep tendon knee jerk reflex. Magnetic resonance imaging (MRI) of the cervical spine revealed a large intradural extramedullary mass located ventral to the spinal cord, localized to the C3 vertebral level, and causing significant spinal cord displacement to the left. Imaging findings were consistent with meningioma as evidenced by the presence of a dural tail, a characteristic radiographic feature (Figure 1). Given the size of the mass and the associated risks, surgical intervention was recommended and discussed with the patient, who consented to the procedure.

A standard right-sided anterior cervical discectomy and fusion approach with neuromonitoring and intraoperative microscopy was utilized. A C3 corpectomy was performed to access the ventral spinal column, and a C2-3 and C3-4 discectomy were performed to determine the cranial-caudal dimensions of the resection cavity, while the uncovertebral joints at these levels were utilized to determine the lateral extent of the exposure. The ventral portion of the C3 vertebral body was removed, and the excised bone was preserved for autograft placement within the cage. A midline durotomy was created, the dura was elevated with dural sutures, and the arachnoid plane caudal to the tumor was visualized. The tumor adherent to the ventral dura was separated, and a small piece of this specimen was sent for pathologic analysis. The Cavitron Ultrasonic Surgical Aspirator (CUSA, Integra, Princeton, NJ) was used to gently and internally debulk the tumor, also allowing for separation of the tumor capsule from the spinal cord and thereby avoiding undue cord compression. The tumor had no vascular attachments beyond its dural base. A 3 Rhoton Micro Dissector was used to detach the remaining tumor from the dura, and the excised portion was removed. By preserving the attached dura, a Simpson Grade II resection was achieved. The surgical field was irrigated, then closed using a running, locking DuraStat (DuraStat, Austin, TX) suture system, which was particularly beneficial given the narrow confines of the surgical corridor.

**Figure 2:**
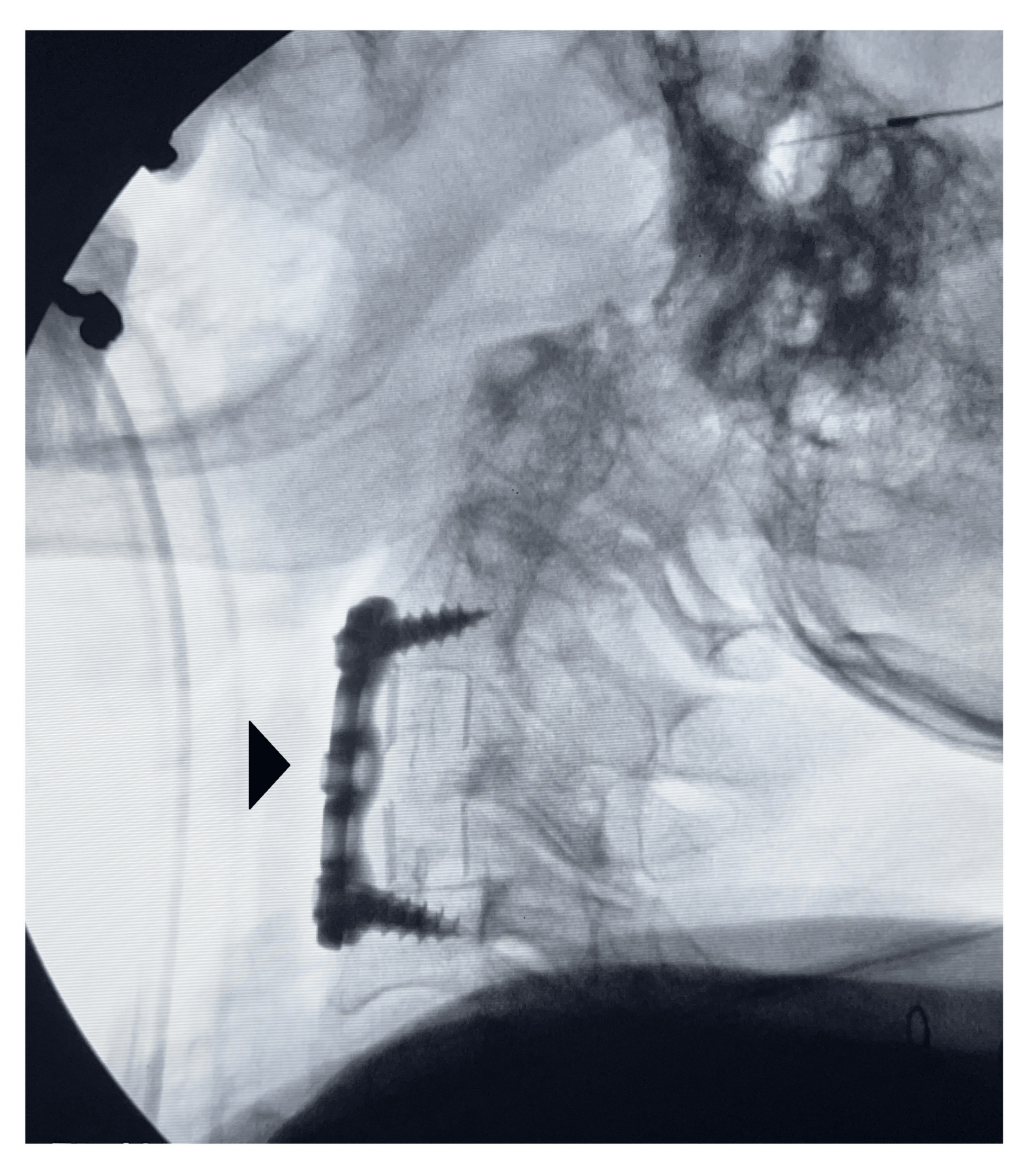
Intraoperative lateral fluoroscopy The arrow demonstrates the anteriorly located titanium plate and screws, with the PEEK cage located posteriorly. PEEK: polyetheretherketone

After tumor resection and dural closure were complete, spinal reconstruction was begun. An autograft from the corpectomy, in addition to Grafton DBM (Medtronic, Minneapolis, MN), was placed within a PEEK cage. PEEK was specifically chosen to minimize artifacts on imaging. A Cornerstone PSR (Medtronic, Minneapolis, MN) strut (22 mm H × 14 mm W × 11 mm D), with positioning confirmed via lateral fluoroscopy. An Atlantis Elite (Medtronic, Minneapolis, MN) 32 mm titanium plate was placed with 4 mm × 13 mm variable screws bilaterally at C2 and 4 mm × 13 mm fixed bilaterally at C4. Final anteroposterior and lateral radiographs were obtained to verify implant placement (Figure 2).

Postoperatively, the patient experienced mild swallowing difficulty, which was managed with steroids and resolved within a short duration. As of the most recent follow-up, two years postoperatively, she has no evidence of residual or recurrent disease, and her myelopathy has resolved.

## Discussion

Spinal meningiomas constitute 25-30% of intradural extramedullary lesions but only 1.2-12% of all meningiomas, predominantly arising in the thoracic spine (60-80%), followed by cervical (15-25%) and lumbar (5-10%) regions, with 15-20% positioned ventral to the cord and the remainder dorsal or dorsolateral [1,5]. Effective resection of these masses poses a challenge due to the variability in tumor location and degree of cord compression. While benign, these tumors may nonetheless carry difficulties due to limited surgical access and a heightened risk of neurological compromise. Subtotal resection of meningiomas increases the risk of recurrence, with 5-10% of lesions recurring within five years postoperatively [3]. Therefore, the goal is maximum safe resection while preserving neurologic function. The anterior approach with corpectomy selected in this case provided excellent access to this ventrally located cervical tumor.

Risks of the anterior approach to the cervical spine include hoarseness and difficulty swallowing [6]. We utilized two Trimline retractors (Artisan, Medford, NJ): one medial-lateral, as normally used, and one cranial-caudal, providing excellent exposure to the surgical cavity and tumor. Our patient did have mild difficulty swallowing immediately after surgery; her dysphagia was treated with a puree diet and postoperative steroids, and at her first office visit two weeks after surgery, she had resumed a regular diet. Despite a right anterior neck approach, preferred by this right-handed surgeon of record, there was no vocal cord paralysis or hoarseness postoperatively.

Cerebrospinal fluid (CSF) leak is another concern after intradural resection of spinal tumors. Such leaks post-durotomy can lead to complications such as pseudomeningocele, infection, dysphagia, or delayed wound healing, all compounding patient morbidity [7]. In this case, robust dural repair was undertaken using DuraStat, a dural suture system, and Duraseal, a dural sealant. The DuraStat system was chosen for its small needle, integrated knot pusher, and small nylon, which make it suitable for establishing a running locking suture. This ensured meticulous primary apposition of the dural defect, while Duraseal established a watertight barrier resistant to CSF pressure. This method of closure proved effective, as no postoperative leak was discovered.

Implanted hardware for the surgical treatment of spinal tumors must display an overlap of several properties. The material must, of course, be biocompatible, nontoxic, and durable enough to withstand the varied stresses of the functional human spine. In the context of instrumentation for spinal neoplasms, the prospective implant should be constructed of a material that minimizes radiologic artifacting, a potentially confounding variable for post-surgical monitoring for tumor recurrence [4,8]. Spinal meningiomas have a roughly 6% recurrence rate at five years post-surgical resection, and resection remains the standard of care for these recurrent tumors, necessarily subjecting the patient again to the risks of spine surgery [3]. The list of options from which to select an implant is not insignificant; however, no material is without its disadvantages, and a careful cost-benefit analysis must be conducted on a patient-by-patient basis [7].

A bone graft provides the advantages of high fusion rates and relative cost-effectiveness but poses the risks of infection, pseudoarthrosis, and immune rejection by the host [9]. Similarly inexpensive is bone cement, but it carries the risks of graft dislodgement, thermal damage to the spinal cord and other surrounding tissues, and esophageal perforation [7]. A 3D-printed spinal implant offers perfect customization and precise replication of complex geometries, resulting in incredibly low rates of subsidence and pseudoarthrosis. However, this burgeoning technology carries with it the expense of a new state-of-the-art device [9]. Lastly, while the current and long-standing gold standard, cages made of titanium-aluminum alloys, produce a degree of artifact sufficient to interfere with radiographic surveillance for meningioma recurrence [6]. For these reasons, a carbon-PEEK implant was selected for vertebral body replacement. Carbon-PEEK implants have been established to have an equivalent safety profile to titanium instrumentation in the thoracic and lumbar spine, and recent data show a similar performance in the cervical spine [9]. PEEK is well-suited to tolerating the mechanical stresses placed on the cervical spine, resists oxidative degradation, and is radiolucent, an essential property in this case and the chief non-structural reason for its selection for vertebral body replacement [6].

## Conclusions

This case of a 73-year-old female undergoing anterior resection of a ventral C3 meningioma demonstrates the intricate interplay of surgical strategy, anatomical precision, and material innovation required to address intradural spinal neoplasms. The approach facilitated maximal safe resection with a Simpson Grade II outcome, underscoring the necessity of tailored approaches in balancing tumor eradication with functional preservation, while the PEEK implant mitigated radiologic artifacting, a critical goal for monitoring tumor recurrence. Fastidious surgical technique minimized blood loss and cord manipulation and prevented the common hazard of CSF leakage, ensuring an uncomplicated postoperative course with transient dysphagia resolving swiftly. By leveraging PEEK's biomechanical resilience and radiolucency over alternative solutions, this intervention not only restored structural integrity but also optimized long-term surveillance. This case reaffirms the utility of the anterior corridor for resecting ventral cervical masses and highlights the pivotal role of advanced biomaterials in enhancing outcomes amidst the challenges of spinal oncology.
